# Supplementary material for: Seminal Extracellular Vesicles and Their Involvement in Male (In)Fertility: A Systematic Review
Source: Int J Mol Sci. 2023 Mar 2;24(5):4818. doi: 10.3390/ijms24054818 (PMC10002921; doi:10.3390/ijms24054818)
Supplement: Supplementary file 1 [file ijms-24-04818-s001.zip › Supplementary file S1.pdf]

<u>Search strategy</u>:

Embase

Terms to query:

('epididymosome':ab,ti OR 'prostasome':ab,ti OR 'exosome':ab,ti OR 'extracellular vesicles':ab,ti OR 'microvesicle':ab,ti)

AND

('semen':ab,ti OR 'seminal plasma':ab,ti OR 'epididymis':ab,ti OR 'ejaculate':ab,ti OR 'sperm':ab,ti OR 'spermatozoa':ab,ti)

PubMed/MEDLINE

Terms to query:

(exosome[Title/Abstract]) OR (extracellular vesicles[Title/Abstract]) OR (microvesicle[Title/Abstract]) OR (prostasome[Title/Abstract]) OR (epididymosome[Title/Abstract])

AND

(semen[Title/Abstract]) OR (seminal plasma[Title/Abstract]) OR (epididymis[Title/Abstract]) OR (ejaculate[Title/Abstract]) OR (sperm[Title/Abstract]) OR (spermatozoa[Title/Abstract])

Scopus

Terms to query:

(TITLE-ABS-KEY (exosome) OR TITLE-ABS-KEY (extracellular AND vesicle) OR TITLE-ABS-KEY (microvesicle) OR TITLE-ABS-KEY (epididymosome) OR TITLE-ABS-KEY (prostasome)

AND

TITLE-ABS-KEY (semen) OR TITLE-ABS-KEY (seminal AND plasma) OR TITLE-ABS-KEY (epididymis) OR TITLE-ABS-KEY (ejaculate) OR TITLE-ABS-KEY (sperm) OR TITLE-ABS-KEY (spermatozoa))
