# Supplementary material for: Seminal Extracellular Vesicles and Their Involvement in Male (In)Fertility: A Systematic Review
Source: Int J Mol Sci. 2023 Mar 2;24(5):4818. doi: 10.3390/ijms24054818 (PMC10002921; doi:10.3390/ijms24054818)
Supplement: Supplementary file 1 [file ijms-24-04818-s001.zip › Supplementary file S4.pdf]

**Supplementary file S4.** Articles initially included in the systematic review because they performed experiments linking seminal extracellular vesicles (sEVs) to fertility outcomes, but ultimately excluded because they did not isolate or adequately characterize sEVs.

| Authors | Species | DOI | Isolation method | Characterization |
|---|---|---|---|---|
| Allegrucci et al. (2001) | Human | 10.1034/j.1600-0897.2001.d01-4.x | UC (x2) + SEC | Not done |
| Andrews et al. (2015) | Human | 10.1093/molehr/gav049 | UC | Not done |
| Carlsson et al. (2004) | Human | 10.1111/j.1365-2605.2004.00468.x | UC (x2) + SEC | Not done |
| Carlsson et al. (2004) | Human | 10.1111/j.1365-2605.2004.00458.x | UC (x2) + SEC | Not done |
| Carlsson et al. (2004) | Human | 10.1002/j.1939-4640.2004.tb02844.x | UC (x2) + SEC | Not done |
| Irazusta et al. (2004) | Human | 10.1002/j.1939-4640.2004.tb02848.x | UC (x2) | Not done |
| Liu et al. (2019) | Rat | 10.1021/acs.jafc.9b01114 | Not done | Not done |
| Lwaleed et al. (2005) | Human | 10.1160/TH04-09-0600 | UC (x2) | Not done |
| Lwaleed et al. (2005) | Human | 10.1111/j.1365-2605.2005.00608.x | UC (x2) | Not done |
| Ronquist et al. (1988) | Human | 10.1159/000281325 | UC | Not done |
| Ronquist et al. (1985) | Human | 10.3109/01485018508986887 | UC | Not done |
| Trigg et al. (2021) | Mouse | 10.1016/j.celrep.2021.109787 | Filtration + gradient UC (x2) | Not done |
| Zucchi et al. (2006) | Human | 10.1016/j.fertnstert.2005.07.1293 | UC (x2) + SEC | Not done |
| Panner Selvam et al. (2019) | Human | 10.5534/WJMH.180108 | Not done | EV specific markers (WB: CD63, ANXA2, TF, KIF5B, SEMG1) |

**ANXA2** (Annexin A2), **sEV** (seminal extracellular vesicles), **KIF5B** (Kinesin Family Member 5B), **SEC** (size-exclusion chromatography), **SEMG1** (Semenogelin-1), **TF** (tissue factor), **TSG101** (Tumor Susceptibility Gene 101), **UC** (ultracentrifugation), **WB** (western blot).
